# Effect of OpunDia^TM^ (O. ficus-indica extract) on oral glucose tolerance and plasma insulin before and after exercise

**DOI:** 10.1186/1550-2783-8-S1-P17

**Published:** 2011-11-07

**Authors:** Ivo Pischel, Karen Van Proeyen, Peter Hespel

**Affiliations:** 1 PhytoLab GmbH & Co. KG, Vestenbergsgreuth, Germany; 2 Research Center for Exercise and Health, Department of Biomedical Kinesiology, Faculty of Kinesiology and Rehabilitation Sciences, K.U. Leuven, Belgium

## Background

High-intensity exercise typically leads to a depletion of body carbohydrate stores, primarily muscle glycogen. Therefore, typical ‘sports recovery drinks’ include a high carbohydrate dose, which stimulate muscle glucose uptake and glycogen re-synthesis via increased plasma insulin level. Thus, interventions that elevate plasma insulin following exercise could facilitate repletion of muscle glycogen stores, and serve as a useful ‘recovery agent’. There are some indications that extracts of the prickly pear cactus (Opuntiaficus-indica; OFI) can stimulate insulin secretion.

## Methods

A double-blind randomized cross-over study was performed. Six subjects participated in two experimental sessions after a 10-12 hr overnight fast with a 2-week interval in between. They received either 1000 mg of encapsulated OFI-extract (OpunDia^TM^, an aqueous extract of OFI; Finzelberg GmbH & Co. KG, Germany), or placebo capsules (LUVOS Heilerde) with identical appearance.

Thirty min after ingestion a 2-hr oral glucose tolerance test (OGTT: 75g of glucose in 300ml water; blood samples (5ml) at 0, 30, 60, 90, and 120 min) was started. Plasma samples were assayed for glucose and insulin concentration. Immediately after this OGTT the subjects performed a cycling exercise bout on an electromagnetically braked bicycle ergometer (Avantronic Cyclus 2, Leipzig, Germany). Following a 10-min warming up (5min @ 60 Watt + 5min @ 120 Watt), they cycled for 30min at a ~70% workload of VO2max. After this exercise bout they received another dose of either 1000 mg of encapsulated OFI-extract, or placebo capsules. Then a second 2-hr OGTT started. However, in this OGTT a dual glucose bolus was administered (75g glucose in 300 ml at time 0 and at time 60 min). Student's paired T-tests were used to evaluate treatment effects. A probability level (p< 0.05) was considered statistically significant.

## Results

Compared with placebo, the area under the blood glucose curve (AUC) was decreased by ~30% after oral administration of OFI, before as well as after exercise (p<0.05). However, AUC for serum insulin was not different between the treatments either before (p= 0.78) or after (p=0.35) exercise. After 60 min of both the basal and the post-exercise OGTT, the intake of OFI reduced blood glucose level by ~10% (p<0.05). During the basal OGTT, initial serum insulin concentration was increased by OFI and remained higher at 30 min in the OGTT(p<0.05). Despite ~15% greater insulin concentrations after OFI ingestion compared with placebo at 30 min and 90 min during the post-exercise OGTT, no statistical significance was reached (p=0.22).

## Conclusion

It was shown that the aqueous extract of OFI can stimulate insulin secretion before and after endurance exercise bouts (although not significant) and lowered the blood glucose level in sportsmen. The aqueous extract of prickly pear (OpunDia^TM^) is a promising and safe ingredient for the development of dietary and sports supplements with anti-hyperglycemic and potential insulin secreting activity. Thus, OpunDia^TM ^might act as a “recovery agent”. Additional studies including muscle biopsies were initiated to test the hypothesis that ingestion of OFI-extract together with carbohydrates can stimulate post-exercise muscle glycogen resynthesis indeed.

